# Effect of TTP488 in patients with mild to moderate Alzheimer’s disease

**DOI:** 10.1186/1471-2377-14-12

**Published:** 2014-01-15

**Authors:** Aaron H Burstein, Imogene Grimes, Douglas R Galasko, Paul S Aisen, Marwan Sabbagh, Adnan MM Mjalli

**Affiliations:** 1 TransTech Pharma, High Point, NC, USA; 2 Department of Neurosciences, University of California, San Diego, CA, USA; 3 Banner Sun Health Research Institute, Sun City, AZ, USA; 4 University of Arizona, Tucson, Arizona, AZ, USA

## Abstract

**Background:**

TTP488, an antagonist at the Receptor for Advanced Glycation End products, was evaluated as a potential treatment for patients with mild-to-moderate Alzheimer’s disease (AD). A previous report describes decreased decline in ADAS-cog (delta = 3.1, p = 0.008 at 18 months, ANCOVA with multiple imputation), relative to placebo, following a 5 mg/day dose of TTP488. Acute, reversible cognitive worsening was seen with a 20 mg/day dose. The present study further evaluates the efficacy of TTP488 by subgroup analyses based on disease severity and concentration effect analysis.

**Methods:**

399 patients were randomized to one of two oral TTP488 doses (60 mg for 6 days followed by 20 mg/day; 15 mg for 6 days followed by 5 mg/day) or placebo for 18 months. Pre-specified primary analysis, using an ITT population, was on the ADAS-cog11. Secondary analyses included as a key secondary variable the Clinical Dementia Rating-Sum of Boxes (CDR-SB), and another secondary variable of the ADCS-ADL.

**Results:**

On-treatment analysis demonstrated numerical differences favoring 5 mg/day over placebo, with nominal significance at Month 18 (delta = 2.7, p = 0.03). Patients with mild AD, whether defined by MMSE or ADAS-cog, demonstrated significant differences favoring 5 mg/day on ADAS-cog and trends on CDR-sb and ADCS-ADL at Month 18. TTP488 plasma concentrations of 7.6-16.8 ng/mL were associated with a decreased decline in ADAS-cog over time compared to placebo. Worsening on the ADAS-cog relative to placebo was evident at 46.8-167.0 ng/mL.

**Conclusions:**

Results of these analyses support further investigation of 5 mg/day in future Phase 3 trials in patients with mild AD.

## Background

Alzheimer’s Disease (AD) is a neurodegenerative disorder with aspects of inflammatory, metabolic and vascular pathology [1,2]. An overproduction of amyloid beta (Aβ) has been implicated as the leading mechanistic factor in AD pathology. Aβ is known to bind to The Receptor for Advanced Glycation Endproducts (RAGE) an immunoglobulin supergene family member expressed on multiple cell types in the brain and the periphery [3,4]. RAGE is found on the cells of the neurovascular compartment: endothelial cells and microglia prominently express RAGE whose expression is upregulated in AD [5,6]. RAGE ligands include Aβ, S100b, HMGB1 and Advanced Glycation Endproducts. RAGE-ligand interactions lead to sustained inflammatory states that play a role in chronic diseases such as diabetes, inflammation, and AD [7,8]. In AD, RAGE has been proposed to contribute to AD pathology by: promoting vascular leakage, promoting influx of peripheral Aβ into brain; mediating Aβ-induced oxidative stress and Aβ mediated neuronal death [9-12].

The pleiotropic role of RAGE has been demonstrated in AD pathology has been described using rodent models. Mice expressing the human APP transgene in neurons develop significant biochemical and behavioral changes reminiscent of human AD. Double transgenic mouse overexpressing WT RAGE in the APP transgene background exhibit accelerated behavioral changes whereas double transgenic animals expressing a dominant negative mutant of RAGE are protected [13]. This data suggests that RAGE plays a role in augmenting the chronic inflammatory state caused by overproduction of Aβ.

RAGE is thought to be involved in the transport of Aβ from peripheral to CNS compartments [14]. In vivo, Aβ uptake into brain is dependent on RAGE as shown in RAGE null mice [12]. Similarly, Aβ uptake in brain can be inhibited using either the secreted, soluble form of RAGE (called sRAGE) or an anti-RAGE antibody [12]. In addition, plaque formation in a mouse model of cerebral amyloidosis was inhibited using sRAGE [15,16]. These data suggest that RAGE is intimately involved in the pathogenesis of AD, and that sustained Aβ interaction with RAGE on blood brain barrier (BBB) and/or neuronal cells is an important element of amyloid plaque formation and chronic neuronal dysfunction.

TransTech Pharma, Inc. discovered TTP488, an orally active, centrally acting antagonist of RAGE-RAGE ligand interaction. Chronic oral dosing of TTP488 in AD transgenic mice led to a reduction of amyloid load in the brain, improved performance on behavioral testing and normalization of electrophysiological recordings from hippocampal slices [17].

**Figure 1:**
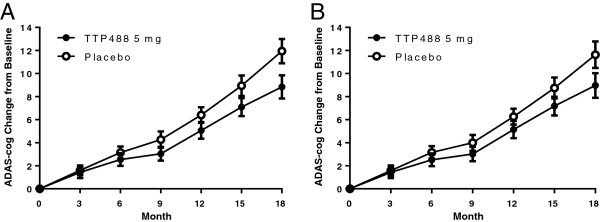
**Estimated mean change from baseline over time on Alzheimer’s Disease Assessment Scale-Cognitive subscale (ADAS-cog 11).** Increasing values represent worsening. Error bars represent one standard error. **(A)** ADAS-cog Observed cases all data. Treatment-placebo difference at 18 months = 3.1, p < 0.008; **(B)** ADAS-cog, on treatment observed cases. Treatment-placebo difference at 18 months = 2.7, p = 0.03.

The results of a phase 2 study examining the safety, tolerability and efficacy of TTP488 in mild to moderate AD have been reported elsewhere [18]. Briefly, 399 patients were randomly assigned to one of two dose levels of TTP488 (60 mg loading dose for 6 days followed by 20 mg/day; 15 mg loading dose for 6 days followed by 5 mg/day) or placebo administered orally for 18 months. The pre-specified primary analysis, using a modified intent-to-treat population, was on the Alzheimer’s Disease Assessment Scale-Cognitive (ADAS-cog11). Based on a pre-specified interim analysis when 50% of subjects had completed the 6 month visit, the 20 mg/day dose was discontinued due to an increased incidence of confusion, falls and greater ADAS-cog decline than placebo. No safety concerns were noted for the 5 mg/day group. Approximately 12 months after all subjects were randomized a second pre-specified interim analysis on 18-month completers compared the 5 mg/day dose and placebo groups for futility and safety. While safety data raised no concerns, the criterion for futility (less than 10% conditional power to observe a significant difference between low dose and placebo at 18 months) was met and treatment was discontinued. Final analysis showed a decreased decline on the ADAS-cog in the 5 mg/day group at month 18 (treatment-placebo difference = 3.1, p = 0.008, ANCOVA with multiple imputation) (Figure 1, panel A). The difference remained significant using other planned statistical models that cope with missing data differently (ANCOVA on observed cases (p = 0.02), ANCOVA with LOCF (p = 0.03), mixed-models repeated measures (p = 0.04), and GEE (p = 0.03)). The authors concluded that this post-futility analysis suggested benefit for 5 mg/day; however, definitive conclusions about the effects could not be made due to operational issues (dropouts and discontinuations from treatment) subsequent to the interim analysis.

This manuscript describes analyses of the effect of TTP488 5 mg/day, versus placebo, on the Alzheimer’s Disease Assessment Scale-Cognitive (ADAS-cog) [19] after 18 months of treatment, in patients on stable background therapy with acetylcholinesterase inhibitors and/or memantine, based on an “on-treatment” definition of the study population. Additionally, analysis of the ADAS-cog, Clinical Dementia Rating Sum of Boxes (CDR-sb) [20] and the Alzheimer’s Disease Cooperative Study – Activities of Daily Living scale (ADCS-ADL) [21] for mild sub-population and characterization of the pharmacokinetic/pharmacodynamic relationship of TTP488 to ADAS-cog are described.

## Methods

This Phase 2, multicenter, randomized, double-blind, placebo-controlled, parallel, three-arm, multiple dose study was conducted at 40 study sites in the United States between January 2007 and December 2010 (ClinicalTrials.gov identifier NCT00566397.) The study was approved by each study site’s Local Institutional Review Board (see Additional file 1). Each patient provided written informed consent. If patients had impaired decisional capacity, caregivers provided consent and patients provided assent.

### Patients

Eligible patients were aged ≥ 50; met the criteria for a diagnosis of probable AD [22]; had a MMSE [23] score between 14 and 26, had a modified Hachinski (Rosen) score ≤4, were receiving treatment with a stable dose of an acetylcholinesterase inhibitor and/or memantine for ≥ 4 months prior to randomization. Patients were excluded for clinically significant neurologic, psychiatric or other diseases contributing to his/her dementia, MRI and/or CT evidence of stroke or significant cerebrovascular disease, uncontrolled hypertension, unstable cardiac or pulmonary disease, diabetes (or hemoglobin A1c at screening > 6%).

### Study design and treatment

Enrollment targeted 399 patients (133 per group), randomized (1:1:1) to placebo, or TTP488 20 mg daily (after a loading dose of 60 mg daily for 6 days), or 5 mg daily (after a loading dose of 15 mg daily for 6 days), for 18 months. An independent Data and Safety Monitoring Board (DSMB) monitored the safety of subjects in the trial.

Study visits occurred at screening, baseline, then at four weeks, 3, 6, 9, 12, 15, 18 months, with a safety follow-up visit at 21 months. Visits included clinical and safety evaluations, blood draw for plasma biomarker and pharmacokinetic analysis, and pill counts to assess compliance. Brain MRIs were obtained at baseline, 12 and 18 months. Lumbar punctures for CSF biomarkers were performed at baseline and 12 months on a subgroup of subjects.

### Outcome measure

The primary efficacy measure was the ADAS-cog [21]. The ADAS-cog/12-item (scored 0–80) scale was administered before the first dose, and at 3, 6, 9, 12, 15 and 18 months with the pre-specified analyses being on the ADAS-cog/11-item scale (Scored 0–70). The key secondary clinical measure was the CDR-sb [22]. The ADCS-ADL was included as a secondary measure [23]. Both CDR-sb and ADCS-ADL were administered prior to dosing and at months 6, 12 and 18.

### Pharmacokinetic assessments

Blood samples for TTP488 PK analysis were collected prior to dosing at Week 1, at Months 1, 3, 6, 9, 12, 15, 18, and 21 and at Early Termination.

### Statistical analysis

#### Populations

The full analysis set (FAS) consisted of all subjects who received at least 1 dose of study medication, and had a baseline (if applicable for the endpoint being analyzed) and post-baseline observation for the measurement of interest. The results of this analysis have been presented previously [18]. The on-treatment analysis set was defined as all available on treatment data, where “on treatment” was defined as date of last dose plus 45 days (this definition was used because the drug has a long ½ life of 10–20 days).

### Primary analysis

The primary analysis planned in the study protocol compared differences in mean treatment effect using 5 statistical methodologies that cope with missing data in different ways, with multiple-imputation methods demarked as primary and others as supportive (supportive methods included endpoint analysis, observed cases, generalized estimating equations (GEE), and mixed-models repeated measures on the longitudinal data). Point estimates, standard errors, confidence intervals, and p-values were computed using the statistical models as planned. For all analyses alpha = 0.05, as the supportive analyses were planned to ensure robustness against missing data.

Baseline measures of the variable of analysis are recommended covariables for statistical modeling [24]. Subgroup analysis for covariables of baseline severity of AD can be based on MMSE or ADAS-cog, the latter of which is the variable of analysis. Use of the baseline ADAS-cog can reduce heterogeneity, thereby increasing the sensitivity of detecting delineation between treatments.

### Pharmacokinetic/pharmacodynamic analyses

Blood samples (5 mL) for TTP488 plasma concentrations were collected in dipotassium (K2) EDTA tubes prior to dosing at Week 1, months 1, 3, 6, 9, 12, 15, 18, 21 and at Early Termination. Samples were centrifuged at approximately 1700 g for about 10 minutes at 4°C with plasma stored in polypropylene tubes at approximately -20°C within 1 hour of collection. Plasma samples were analyzed for TTP488 concentrations using a validated HPLC-MS/MS method.

Exploratory analyses relating TTP488 plasma concentration (including the 20 mg/day and 5 mg/day dose groups) to ADAS-cog values, and changes over time, utilized deriving a subject-level value by two methods: (1) deriving the subject level value by the maximum of the trough concentration values for that subject over the 18-month period, and (2) deriving the subject level value by taking the median concentration value for that individual. Analyses were done at the subject level.

Subjects were classified into concentration groups according to quintile cut-points in the distribution of concentration values ignoring administered dose.

### Analysis of mild vs. moderate subgroups

Protocol-planned analyses included subgroup analysis based on baseline severity of AD. ADAS-cog and CDR-sb changes from baseline were analyzed (ANCOVA adjusted for baseline main effects) by baseline disease severity using an MMSE based definition of mild AD (MMSE ≥ 20). ADAS-cog, CDR-sb and ADCS-ADL were additionally evaluated (2-sample t-test) using an ADAS-cog based definition of mild AD (ADAS-cog ≤ 19). An ADAS-cog value of 19 was selected based on conversion of a traditional cut-off of an MMSE value of 20, used in the analyses reported above, to a corresponding ADAS-cog value using the previously described linear relationship between ADAS-cog and MMSE (ADAS-cog = 56.4-1.86*MMSE) [25].

### Sample size

With approximately 133 subjects per group, the primary study had 80% power to detect a 3 point difference in change from baseline to 18 months in ADAS-cog scores between a TTP488 dose group and placebo, allowing for 25% missing data and two interim analyses. ADAS-cog 18- month changes from baseline were assumed to have a standard deviation of 6.5 points for all treatment groups. A total experiment-wise Type 1 error rate of α= 0.05 was targeted.

## Results

### Patient disposition

Patient disposition has been described previously [18]. Briefly, 701 subjects were screened for eligibility, 302 were excluded and 399 were randomized. Subjects were well matched for demographic characteristics with no significant differences between groups (Table 1).

**Table 1:** Patient demographic and baseline clinical characteristics (including all on- and off-drug data)

	**TTP488 20 mg/day**	**TTP488 5 mg/day**	**Placebo**
	**(n = 135)**	**(n = 131)**	**(n = 133)**
Age (years)	73.0 ± 9.0	73.6 ± 8.8	72.2 ± 9.6
Sex (% women)	61	53	57
Race			
White	128	120	125
Education (years)	15.0 ± 3.0	14.8 ± 2.8	15.3 ± 2.8
MMSE	19.9 ± 3.6	20.8 ± 3.5	20.5 ± 3.4
Mild (MMSE ≥20), n(%)	71 (53%)	84 (64%)	81 (61%)
Moderate (MMSE < 20), n(%)	64 (47%)	47 (36%)	51 (39%)
ADAS-cog	24.9 ± 9.7	24.4 ± 9.8	24.1 ± 9.6
CDR-sb	5.7 ± 2.9	5.6 ± 2.7	6.0 ± 2.8
ADCS-ADL	61.3 ± 12.9	61.4 ± 12.3	59.9 ± 12.8
NPI	7.9 ± 10.5	7.7 ± 10.3	8.6 ± 10.4
APOE e4+status, n (%)	62	65	74
E2/E3	3 (2.3%)	5 (4.2%)	1 (0.9%)
E2/E4	7 (5.4%)	2 (1.7%)	2 (1.7%)
E3/E3	46 (35.7%)	36 (30.5%)	30 (25.6%)
E3/E4	47 (36.4%)	53 (44.9%)	59 (50.4%)
E4/E4	26 (20.2%)	20 (16.9%)	25 (21.4%)
Unknown/Missing	6	15	16
AchEI use, n(%)	134 (99%)	129 (98%)	132 (100%)
Memantine use, n (%)	92 (68%)	87 (66%)	96 (73%)

Unless otherwise indicated, data reported as mean ± SD.

MMSE = Mini-Mental State Examination, ADAS-cog = Alzheimer’s Disease Assessment Scale cognitive. CDR-sb = Clinical Dementia Rating sum of boxes, ADCS-ADL = Alzheimer’s Disease Cooperative Study Activities of Daily Living Scale, NPI = Neuropsychiatric Inventory, APOE e4 = Apolipoprotein E e4 allele, AchEI = acetylcholinesterase inhibitor.

### On-treatment analysis

Statistical analysis on ADAS-cog was performed using all available on-treatment data. Beginning with Month 3, there were 127 subjects in the placebo group and 126 subjects in the 5 mg/day dose group with baseline and on-treatment data. Mean changes and median changes in ADAS-cog are consistent in showing numerical active-placebo differences favoring the 5 mg/day dose group over time. (Table 2, Figure 1, panel B) At all time points, the numerical difference favors the 5 mg/day dose group over placebo, with nominal significance at Month 18 (Δ=2.7, p = 0.03).

**Table 2:** Changes from baseline in ADAS-cog for on-treatment population

	**3 months**	**6 months**	**9 months**	**12 months**	**15 months**	**18 months**
5 mg/day	1.43 ± 0.50	2.52 ±0.56	3.02 ± 0.62	5.14 ± 0.75	7.18 ± 0.81	8.96 ± 1.07
	(n = 126)	(n = 118)	(n = 106)	(n = 95)	(n = 83)	(n = 63)
Placebo	1.58 ± 0.44	3.16 ± 0.54	3.99 ± 0.68	6.26 ± 0.69	8.74 ± 0.91	11.63 ± 1.15
	(n = 127)	(n = 114)	(n = 109)	(n = 101)	(n = 86)	(n = 59)
						p = 0.03

Data presented as mean ± standard error.

### Exploratory analysis by disease severity

**Figure 2:**
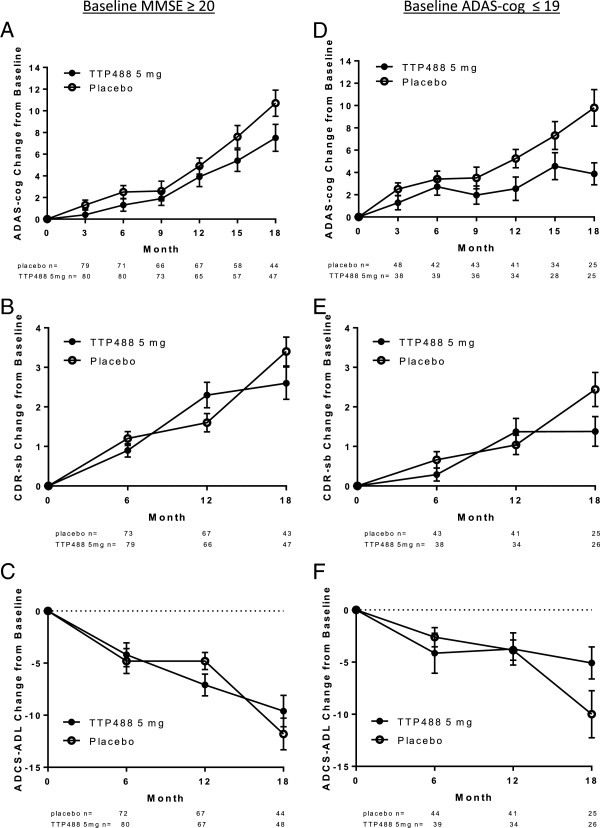
**Estimated mean change from baseline over time on ADAS-cog 11, CDR-sb and ADCS-ADL for patients with mild Alzheimer’s disease defined as either MMSE ≥20 (Panels A, B and C) or ADAS-cog ≤19 (Panels D, E, F) at baseline.** Error bars represent one standard error. **(A)** ADAS-cog treatment-placebo difference at 18 months = 3.3, p = 0.024, Baseline MMSE ≥ 20; **(B)** CDR-sb treatment-placebo difference at 18 months = 0.72, p = 0.053, Baseline MMSE ≥ 20; **(C)** ADCS-ADL treatment – placebo difference at 18 months = 2.2, p = 0. 3, Baseline MMSE ≥ 20 **(D)** ADAS-cog treatment-placebo difference at 18 months = 5.9, p < 0.008, Baseline ADAS-cog ≤ 19; **(E)** CDR-sb treatment-placebo difference at 18 months = 1, p = 0.08, Baseline ADAS-cog ≤ 19; **(F)** ADCS-ADL treatment-placebo difference at 18 months = 4.92, p = 0.07, Baseline ADAS-cog ≤ 19.

Analysis was performed on the FAS (ITT) comparing ADAS-cog and CDR-sb in patients with mild AD (MMSE ≥20) and moderate AD (MMSE < 20)). For ADAS-cog, a 3.3 point (p = 0.024) and 2.7 point (p = 0.4) difference between 5 mg/day and placebo was seen at Month 18 in the mild group and moderate group, respectively. For CDR-sb , a 0.72 point (p = 0.053) and 0.74 point (p = 0.5) difference between 5 mg/day and placebo was seen at Month 18 in the mild group and moderate group, respectively. These findings were confirmed in post-hoc subgroup analyses defining mild patients as having baseline ADAS-cog ≤19 (n = 25 in each treatment group, observed cases at 18 months). These analyses reveal a delta at 18 months, favoring 5 mg/day over placebo, on ADAS-cog of 5.9 points (p < 0.01) and trends on CDR-sb (delta = 1, p = 0.08) and ADCS-ADL (delta = 4.92, p = 0.07) (Figure 2). The beneficial effect of 5 mg/day in patients with moderate AD (ADAS-cog >19) was less pronounced with a delta (5 mg/day vs. placebo) at 18 months of 1.45 for ADAS-cog, 0.74 for CDR-sb, and 0.57 for ADCS-ADL.

### TTP488 Plasma concentration driven analysis

**Figure 3:**
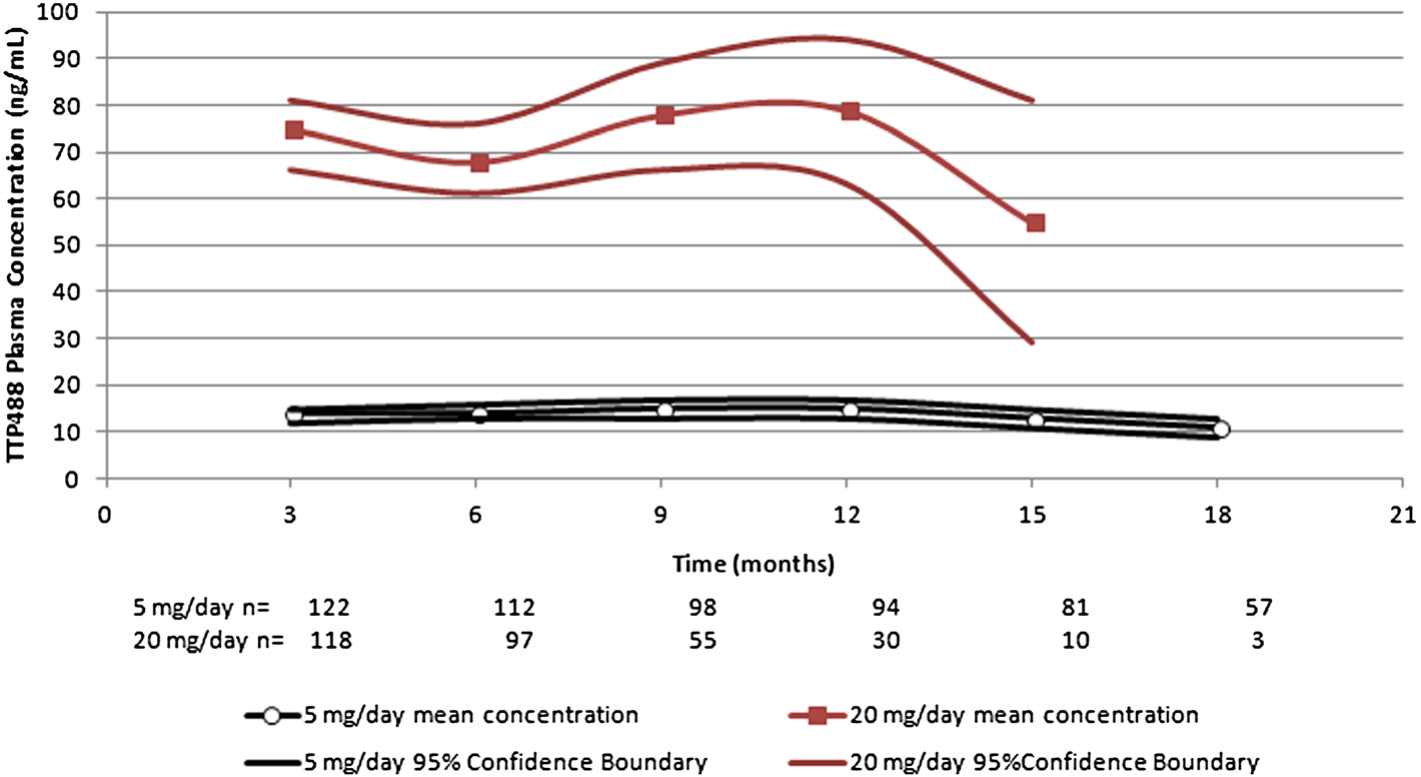
**TTP488 plasma concentration over time.** On-treatment data where on-treatment is defined as plasma concentrations measured within 45 days of last administered dose. Data presented as mean values with 95% confidence boundary. For the 20 mg/day group data presented through Month 15 after which there were too few observations.

As expected, higher plasma TTP488 trough concentrations (mean, median of trough concentrations over the study duration, mean of trough concentrations over study duration) were observed for 20 mg/day, and lower concentrations observed for 5 mg/day (Table 3, Figure 3).

**Table 3:** TTP488 median and mean trough concentrations associated with the 5 mg/day and 20 mg/day dose groups

	**TTP488 dose group**	**TTP488 mean concentration**^**a**^	**TTP488 median concentration**^**b**^	**95% confidence interval of the mean**
		**(ng/mL)**	**(ng/mL)**	
Median of subjects’ trough values	5 mg/day (n = 131)	13.02	12.25	[11.74, 14.31]
	20 mg/day (n = 134)	68.57	64.58	[63.46, 73.69]
Mean of subjects’ trough values	5 mg/day (n = 131)	16.22	14.90	[14.59, 17.85]
	20 mg/day (n = 134)	83.75	75.05	[77.40, 90.10]

Mean and median concentration across the measured timepoints was determined for each subject. Descriptive statistics (mean, median) subsequently provided for each measure.

^**a**^Mean TTP488 concentration across the 18 months, or available time points, for each individual subject. Each subject contributes a single value.

^**b**^Median TTP488 concentration across the 18 months, or available time points, for each individual subject. Each subject contributes a single value.

Within certain concentration ranges, delineation from placebo in changes in ADAS-cog was more pronounced than in other ranges (Table 4). Subjects with concentrations in the lowest 20% (0.1-10.2 ng/mL) and second lowest 20% (10.3-16.8 ng/mL) showed benefit over placebo at 18 months. The effect in the 0.1-10.2 ng/mL group was primarily driven by those subjects with concentrations greater than 7.6 ng/mL. Subjects in the middle 20% group (17.0-46.3 ng/mL) showed similar effects as placebo. Subjects in the top two groups (46.8-71.7 ng/mL and 74.0-167.0 ng/mL) showed a numerical worsening in ADAS-cog relative to placebo through 12 months after which time the number of subjects in these quintiles is too few to allow for meaningful interpretations.

**Table 4:** Changes from baseline in ADAS-cog for each quintile TTP488 concentration range over 18 months of treatment

**Month**	**Placebo**	**0.1-10.2 ng/mL**	**10.3-16.8 ng/mL**	**17.0-46.3 ng/mL**	**46.8-71.7 ng/mL**	**75-167 ng/mL**
3	1.59 (0.43)	0.83 (0.75)	1.35 (0.74)	2.51 (0.77)	4.42 (0.84)	7.4 (0.94)
	n = 129	n = 50	n = 51	n = 52	n = 47	n = 48
6	3.14 (0.54)	2.24 (0.85)	2.3 (0.87)	4 (0.88)	4.7 (0.82)	9.1 (1)
	n = 115	n = 48	n = 49	n = 48	n = 37	n = 46
9	4.3 (0.7)	1.9 (0.9)	3 (0.93)	4.3(1.1)	5.1 (1.45)	8.4 (1.3)
	n = 112	n = 39	n = 46	n = 42	n = 27	n = 40
12	6.4 (0.68)	3 (0.76)	5.6 (1.22)	6.7 (1.3)	7.4 (1.67)	9.3 (1.3)
	n = 105	n = 32	n = 42	n = 37	n = 22	n = 31
15	9.9 (0.9)	6.8 (0.98)	6.9 (1.42)	8.1 (0.4)	9.3 (1.97)	10.1 (1.9)
	n = 89	n = 30	n = 37	n = 30	n = 21	n = 24
18	11.9 (1.1)	8.7 (1.36)	8.1 (1.71)	10.1 (2)	10.1 (2.47)	11.3(1.8)
	n = 64	n = 25	n = 26	n = 25	n = 15	n = 19

Data are reported as mean (sd), n.

## Discussion

This Phase 2 trial explored the safety and efficacy of 2 doses levels of TTP488, compared to placebo, in patients with mild-to-moderate AD. Post-futility, protocol-specified analyses of changes in ADAS-cog showed a favorable effect in the 5 mg/day dose group compared to placebo at month 18. Sensitivity analyses using methodologies that cope with missing data differently indicated that conclusions were invariant to statistical model or methodology, thereby supporting the robustness of the result. Post-hoc analyses of subjects “on treatment” also demonstrated significant treatment effects for the 5 mg/day dose group.

Effects on the ADAS and CDR-SB were slightly greater and similar in magnitude, respectively, in mild versus moderate subjects defining each subgroup based on MMSE. Given the MMSE is a brief screening test, analyses based on an ADAS-cog based definition of mild AD (consistent with recommended statistical methodologies for accommodating baseline measures of the variable of analysis as a covariable) provide additional confirmation of the effect of TTP488 in patients with mild AD. The inability to demonstrate a significant effect on the ADAS-cog and CDR-sb, despite clinically meaningful numerical effect sizes, in moderate subjects is presumably due the smaller group size and increased variability. While a 5 mg dose of TTP488 may impart beneficial effects in both mild and moderate subjects, enrichment of a study population for mild subjects may allow for not only a greater magnitude of effect but early demonstration of clinical effects.

The results of analyses of ADAS-cog, based on plasma concentrations suggest 5 mg/day, associated with plasma concentrations of 7.6-16.8 ng/mL, as a dose that would be associated with beneficial effects on cognition relative to placebo. While concentrations above 46.8 ng/mL were associated with reversible worsening of cognition in TTP488 treated subjects, the ability to safely dose subjects in the concentration range of 17.0-46.3 ng/mL provides an adequate safety margin for the 5 mg/day dose thereby allowing for accommodation of increased intersubject variability that may be seen in an expanded Phase 3 population.

## Conclusions

This Phase 2 trial demonstrated a 3.1 point difference in ADAS-cog at 18 months for the 5 mg/day dose relative to placebo in patients with mild-to-moderate AD; an effect supported by “on-treatment” analyses of the data. Secondary analyses evaluating the effect in mild patients demonstrated a significant effect on ADAS-cog and trend on CDR-sb and ADCS-ADL at 18 months. This finding supports the enrichment of future TTP488 trials with mild subjects, to allow for demonstration of effects with a dose of 5 mg/day plus standard of care.

## Competing interests

Aaron Burstein is an employee of TransTech Pharma.

Imogene Grimes is an employee of TransTech Pharma.

Douglas Galasko has received research grants from University of California San Diego, National Institutes of Health, Michael J Fox Foundation, Alzheimer’s Drug Discovery Foundation; has been a consultant for Elan Pharmaceuticals, and has received fees as a DSMB member for Elan Pharmaceuticals, Janssen Immunotherapy, Balance Pharmaceuticals and received fees as an Editor of Alzheimer’s Research and Therapy.

Paul Aisen has received research grants from Lilly, Baxter, NIA, FNIH and has been a consultant and/or received honoraria from NeuroPhage; Elan Corporation, Wyeth, Eisai Inc., Schering-Plough Corp., Bristol-Myers Squibb, Eli Lilly and Company, NeuroPhage, Merck & Co., Roche, Amgen, Genentech, Inc., Abbott, Pfizer Inc, Novartis, Bayer, Astellas, Dainippon, Biomarin, Solvay, Otsuka, Daiichi, AstraZeneca, Janssen, Medivation, Inc., Ichor, Toyama, Lundbeck, Biogen Idec, iPerian, Probiodrug, Somaxon, Biotie, Anavex and Kyowa Hakko Kirin Pharma.

Marwan Sabbagh has received research grants from Pfizer, Eisai, Lilly, Avid, Bristol-Myers Squibb, Avanir, Janssen, Elan, Bayer, Paramal, Genentech is a consultant to TransTech Pharma.

Adnan Mjalli is an employee of TransTech Pharma.

## Authors’ contributions

DG, PA and MS were involved in the concept, design and conduct of this study and reviewed the content of the manuscript. AB, IG, AM were involved in the analysis and interpretation of secondary analyses described in this manuscript. AB was responsible for drafting of the manuscript. All authors have reviewed, read and approved the final version of the manuscript for submission to BMC Neurology.

## Pre-publication history

The pre-publication history for this paper can be accessed here:

http://www.biomedcentral.com/1471-2377/14/12/prepub

## Supplementary Material

Additional file 1 Listing of Investigational Review Boards/Ethics Committees approving the conduct of this study. Click here for file
