# Supplementary material for: Effect of TTP488 in patients with mild to moderate Alzheimer’s disease
Source: BMC Neurol. 2014 Jan 15;14:12. doi: 10.1186/1471-2377-14-12 (PMC4021072; doi:10.1186/1471-2377-14-12)
Supplement: Additional file 1 — Listing of Investigational Review Boards/Ethics Committees approving the conduct of this study. [file 1471-2377-14-12-S1.docx]

**Additional file 1. Listing of Investigational Review Boards / Ethics Committees approving the conduct of this study.**

Banner Health IRB

Sun Health IRB

IRB University of California Irvine

Georgetown University IRB

University of California, San Diego UCSD Human Research Protection Program

New York School of Medicine IRB

Mayo Clinic IRB

Yale Human Investigations Committee

The Cleveland Clinic Foundation IRB

Office of Regulatory Affairs, Hospital of the University of Pennsylvania

University of Washington Human Subject Division

University of Wisconsin-Madison Health Sciences and Minimal Risk IRB

Human Research Protections Office – Washington University in St. Louis

Office for the Protection of Research Subjects

University of Pittsburgh IRB

Columbia Presbyterian Medical Center IRB

Western IRB

Baylor College of Medicine Institutional Review Board for Human Subjects

University of Michigan School Institutional Review Board

Lifespan Institutional Review Board

IUPUI & Clarian IRB

Partners Institutional Review Board

Northwestern University Institutional Review Board

Oregon Health Sciences University IRB

Emory University IRB

Rush IRB

Office of Research Integrity, University of Kentucky

University Hospitals of Cleveland IRB

Institutional Review Board for Human Research Medical University of South Carolina

UC Davis, Office of Research IRB Administration

Stanford IRB

UT Southwestern Medical Center

USF Office of Research, Division of Research Compliance

University of Southern California Health Sciences Institutional Review Board

Meharry Medical College IRB

University of Utah Institutional Review Board for Research with Human Subjects

University of California, Irvine IRB

Institutional Review Board, Howard University Hospital

Mount Sinai Medical Center IRB

UCSF Committee on Human Research

Johns Hopkins Institutional Review Boards

Saint Mary’s Health Care IRB

BioMedical Research Institute of America
